# Supplementary material for: Chitosan–Polyvinyl Alcohol Nanocomposites for Regenerative Therapy
Source: Polymers (Basel). 2023 Dec 1;15(23):4595. doi: 10.3390/polym15234595 (PMC10708655; doi:10.3390/polym15234595)
Supplement: Supplementary file 1 [file polymers-15-04595-s001.zip › polymers-2738250-supplementary.pdf]

Supplementary Materials

Table S1. Volatile compounds identified in *Syzygium aromaticum L.* (clove) essential oil.

Compound	Kovats Index		RT	Relative amount (%)
	Exp.	Lit.		
α-Pinene	935	932 [1]	16.1	*tr.*
Δ^3-Carene	1011	1008 [1]	19.4	*tr.*
Heptanyl-2-acetate	1038	1045 [3]	20.6	*tr.*
2-Nonanone	1091	1092 [3]	22.8	*tr.*
Methyl salicylate	1197	1192 [3]	26.9	0.2
Chavicol	1252	1247 [3]	28.9	0.2
Eugenol	1362	1356 [1]	33.0	76.5
α-Copaene	1384	1374 [1]	33.8	0.1
Methyl eugenol	1400	1402 [3]	34.4	*tr.*
trans-β-Caryophellene	1432	1419 [3]	35.5	4.0
α -humulene	1468	1454 [2]	36.8	0.5
Eugenyl acetate	1519	1521 [1]	38.5	17.8
δ-Cadinene	1526	1522 [1]	38.7	0.1
Caryophyllene oxide	1596	1582 [1]	40.8	0.5
11,11-Dimethyl-4,8-dimethylenebicyle[7.2.0]undecane	1651	1646 [3]	42.3	0.1

References

1. Adams, P. *Identification of Essential Oil Components by Gas Chromatography/Mass Spectrometry*, 4th ed.; Allured Publishing Corporation: Carol Stream, IL, USA, 2004
2. Babushok, V.I.; Linstrom, P.J.; Zenkevich, I.G. Retention Indices for Frequently Reported Compounds of Plant Essential Oils. *J. Phys. Chem.* **2011**, *40*, 1–47.
3. NIST Mass Spectrometry Data Center. Available online: http://webbook.nist.gov/chemistry/ (accessed on 1 October 2021).

Disclaimer/Publisher's Note: The statements, opinions and data contained in all publications are solely those of the individual author(s) and contributor(s) and not of MDPI and/or the editor(s). MDPI and/or the editor(s) disclaim responsibility for any injury to people or property resulting from any ideas, methods, instructions or products referred to in the content.
